# Ohio Lunatic Asylum

**Published:** 1842-03

**Authors:** 


### OHIO LUNATIC ASYLUM.

In the elaborate and well digested article of Dr. Jarvis, (No. 24 of this Journal) ample justice is done to the *Ohio Lunatic Asylum*, by an abstract from its annual reports for 1839 and '40. Since the publication of that review in December, we have receivcd the Report for 1841, which presents evidence of continued success in its management; but, unfortnnately, its benefits can not be extended to all the insane of the State; for, extensive as the edifice is, it appears that from the opening of the institution, Nov. 1838, to the date of this Report, three years, no less than 167 applications, formal and informal, from that State, had been refused, to say nothing of 1 from Wiskonsan, 1 from Alabama, 2 from Iowa, 2 from New York, 2 from Virginia, 4 from Kentucky, 7 from Illinois, 9 from Pennsylvania, and 13 from Indiana, equal to 41; indicating a want of such establishments in the States surrounding Ohio. Such of our brethren, in those States, as may wish to place their patients in the Ohio Asylum, will perceive, then, that it will be in vain to apply. It will be for them to exert their influence at home for the establishment of new asylums. If Indiana would found one, it might, for several years, answer for herself and her younger sister Illinois. We commend it to our readers in that State, as eminently worthy of their utmost efforts.

